# Efficacy and safety of fremanezumab in clinical trial participants aged ≥60 years with episodic or chronic migraine: pooled results from 3 randomized, double-blind, placebo-controlled phase 3 studies

**DOI:** 10.1186/s10194-021-01351-2

**Published:** 2021-11-24

**Authors:** Stephanie J. Nahas, Steffen Naegel, Joshua M. Cohen, Xiaoping Ning, Lindsay Janka, Verena Ramirez Campos, Lynda J. Krasenbaum, Dagny Holle-Lee, David Kudrow, Christian Lampl

**Affiliations:** 1 Department of Neurology, Thomas Jefferson University, Philadelphia, PA USA; 2 Department of Neurology, University Hospital Halle (Saale) and University Halle-Wittenberg, Halle, Germany; 3 Teva Pharmaceutical Industries, West Chester, PA USA; 4 Department of Neurology and Westgerman Headache Center Essen, University Hospital, Essen, Germany; 5 California Medical Clinic for Headache, Santa Monica, CA USA; 6 Headache Medical Centre, Linz, Austria; 7 Department of Neurology, Konventhospital Barmherzige Brüder, Linz, Austria

**Keywords:** Episodic migraine, Chronic migraine, Fremanezumab, CGRP, Older age

## Abstract

**Background:**

Although migraine is less common in older people, preventive treatment of migraine in these individuals may be more challenging due to the presence of multiple comorbidities and polypharmacy. Additionally, evidence for migraine treatment efficacy, safety, and tolerability is limited in this population. We evaluated efficacy, safety, and tolerability of fremanezumab, a fully humanized monoclonal antibody (IgG2Δa) that selectively targets calcitonin gene–related peptide (CGRP), in clinical trial participants aged ≥60 years with episodic migraine (EM) or chronic migraine (CM).

**Methods:**

This analysis included data from 3 randomized, double-blind, placebo-controlled phase 3 studies: the HALO EM study, HALO CM study, and FOCUS study in participants with EM or CM and prior inadequate response to 2–4 migraine preventive medication classes. Participants in all studies were randomized 1:1:1 to receive 12 weeks of subcutaneous treatment with quarterly fremanezumab (Months 1/2/3: EM/CM, 675 mg/placebo/placebo), monthly fremanezumab (Months 1/2/3: EM, 225 mg/225 mg/225 mg; CM, 675 mg/225 mg/225 mg), or matched monthly placebo.

**Results:**

These pooled analyses included 246 participants aged ≥60 years. Reductions in monthly migraine days from baseline over 12 weeks were significantly greater with fremanezumab (least-squares mean change from baseline [standard error]: quarterly fremanezumab, − 4.3 [0.59]; monthly fremanezumab, − 4.6 [0.54]) versus placebo (placebo, − 2.3 [0.57]; both *P* < 0.01 vs placebo). As early as Week 1, significant reductions from baseline in weekly migraine days were observed with fremanezumab versus placebo (both *P* < 0.01). With fremanezumab treatment versus placebo, a significantly higher proportion of participants achieved ≥50% reduction in monthly migraine days, and significant improvements in disability and quality-of-life outcomes were observed (*P* < 0.05). Proportions of participants experiencing serious adverse events and adverse events leading to discontinuation were low and similar in the fremanezumab and placebo groups. Efficacy and safety results were comparable to the overall pooled population (*N* = 2843).

**Conclusions:**

This pooled subgroup analysis demonstrates that fremanezumab treatment is efficacious and well-tolerated over 12 weeks in participants aged ≥60 years with EM or CM. These data may help healthcare providers with clinical decision making and preventive treatment selection for older patients with migraine.

**Trial registration:**

ClinicalTrials.gov identifiers: HALO CM: NCT02621931; HALO EM: NCT02629861; FOCUS: NCT03308968.

**Supplementary Information:**

The online version contains supplementary material available at 10.1186/s10194-021-01351-2.

## Background

Migraine is the second leading cause of years lived with disability globally and is associated with a substantial negative impact on health-related quality of life [1–4]. Although migraine is less common in older people, a high prevalence of psychiatric disorders, such as depression, anxiety, and sleep disturbances, and the presence of multiple comorbidities, such as cardiovascular (CV) disorders and diabetes, may be associated with even further worsening in quality of life [5–9]. For example, individuals with migraine may have a more than 5 times greater risk of developing major depressive disorder compared with those without migraine [10–13], and that increased risk of depression is also observed in older people with migraine [8]. In addition, preventive treatment of migraine in older patients may be more challenging due to polypharmacy, especially with respect to medications used for comorbidities, and concerns around cognitive impairment, influenced by comorbidities, medications, and lifestyle [5, 7].

Treatment for patients with migraine includes both acute and preventive medications. For years, preventive treatment options have included anticonvulsants, antidepressants, antihypertensives (eg, β-blockers), flunarizine, and onabotulinumtoxinA [14, 15]. However, these preventive treatments are not specific to migraine and are often unsatisfactory due to lack of efficacy, intolerability, and poor adherence [16–18]. Some of these may also have contraindications, especially in older patients.

There are currently 4 monoclonal antibodies (mAbs) targeting the calcitonin gene–related peptide (CGRP) pathway that are approved by the US Food and Drug Administration (FDA) for the preventive treatment of migraine [19–22]. Fremanezumab, a fully humanized mAb (IgG2Δa) that selectively targets CGRP, has proven efficacy for the preventive treatment of migraine in adults [23–25]. Three randomized, double-blind, placebo-controlled, phase 3 trials have demonstrated that fremanezumab is well tolerated and efficacious in the preventive treatment of episodic migraine (EM) and chronic migraine (CM), even in individuals with difficult-to-treat migraine [23–25]. Long-term safety and efficacy of fremanezumab treatment was also demonstrated for up to 12 months in parallel-group phase 3 studies in participants with EM or CM [26].

The analyses presented here aim to evaluate the efficacy, safety, and tolerability of fremanezumab in participants ≥60 years of age with EM or CM, which would add to the presently limited body of evidence regarding migraine treatment efficacy, safety, and tolerability in this population [5]. Given the worldwide increase in life expectancy, migraine in older age is likely to become an increasing issue over the next 40 years, with management likely confounded by other health problems and consequent association with polypharmacy. Therefore, these analyses with a selected age cutoff of ≥60 years were performed.

## Methods

### Study design

This was a pooled subgroup analysis including data from 3 international, multicenter, randomized, double-blind, placebo-controlled, parallel-group, phase 3 trials in participants with CM and EM: HALO CM (ClinicalTrials.gov Identifier: NCT02621931), which included participants with CM; HALO EM (ClinicalTrials.gov Identifier: NCT02629861), which included participants with EM; and FOCUS (ClinicalTrials.gov Identifier: NCT03308968), which included participants with CM or EM who had a documented inadequate response or contraindication to 2 to 4 classes of prior migraine preventive medications. Detailed methods and study designs for the HALO and FOCUS studies have been previously reported [23–25] and are briefly summarized here.

### Participant population

#### HALO CM and HALO EM

Participants eligible for the HALO studies included adults (18–70 years of age) with a history of migraine per International Classification of Headache Disorders (ICHD)-3 beta criteria for ≥12 months prior to screening with onset at ≤50 years of age. In the HALO CM study, CM was defined as headache on ≥15 days per month, with ≥8 days fulfilling ICHD-3 beta criteria for migraine, probable migraine, or use of triptan or ergot medications, over a period of 3 months [25]. In the HALO EM study, EM was defined as headache on 6 to 14 days per month, with ≥4 days fulfilling ICHD-3 beta criteria for migraine, probable migraine, or use of triptan or ergot medications [23]. Participants were excluded in both trials for use of onabotulinumtoxinA in the 4 months before screening, use of opioids or barbiturates on ≥4 days per month, use of interventions or devices for migraine in the 2 months before screening, or previous failure from ≥2 medication clusters after ≥3 months of treatment (antiepileptics, calcium channel blockers, antidepressants, beta blockers) [23, 25]. Up to 30% of participants were permitted the use of 1 preventive migraine medication if the dose was stable from ≥2 months before the pretreatment period to the end of the treatment period [23, 25].

#### FOCUS

Eligible participants for the FOCUS study included the same adult population as the HALO studies but with 1 key difference: eligible participants also had documented inadequate response or contraindication within the past 10 years to 2 to 4 of the following classes of prior migraine preventive medications: β-blockers, anticonvulsants, tricyclic antidepressants, calcium channel blockers, angiotensin II receptor antagonists, onabotulinumtoxinA, and valproic acid [24]. An inadequate response was defined as documentation in their medical record of no clinically meaningful improvement (per the treating physician’s judgment) after 3 months of stably dosed treatment or discontinuation due to poor tolerability [24].

### Standard protocol approvals, registrations, and participant consents

All 3 studies were conducted in accordance with their respective study protocols and the International Conference for Harmonisation guidelines for Good Clinical Practice, the Declaration of Helsinki, and relevant national and local regulations. The study protocols were approved by the appropriate ethics committees and institutional review boards. Participants provided written informed consent prior to performing any study procedure or assessment [23–25].

### Study design

All 3 studies included a screening visit, a 28-day pretreatment period, a 12-week treatment period (double-blind and placebo-controlled), and a final evaluation at Week 12. Enrolled participants were randomly assigned 1:1:1 to receive subcutaneous quarterly fremanezumab (Month 1/2/3: 675 mg/placebo/placebo), monthly fremanezumab (Month 1/2/3: CM, 675 mg/225 mg/225 mg; EM, 225 mg/225 mg/225 mg), or matched monthly placebo. Efficacy was evaluated using information entered by participants in a daily electronic headache diary throughout the treatment period [23–25].

### Outcome measures

Post hoc analyses were conducted using these pooled data to assess the efficacy and safety of fremanezumab in a subgroup of participants ≥60 years of age. Efficacy outcomes in the overall pooled population are also presented here for comparison.

Outcome measures assessed in participants ≥60 years included the following: mean change from baseline (28-day pretreatment period) in the monthly average number of migraine days during the 12-week treatment period; monthly average number of headache days of at least moderate severity during the 12-week treatment period; monthly average number of days of any acute headache medication use; and weekly average number of migraine days during the first 4 weeks of treatment.

For this subgroup of participants ≥60 years, the following outcomes were also assessed:

- Proportion of participants with ≥50% reduction from baseline (28-day pretreatment period) in the monthly average number of migraine days during the 12-week treatment period
- Mean change from baseline (Day 0) in scores on the 6-item Headache Impact Test (HIT-6; scores range from 36 to 78, with higher scores indicating greater impact of headache on functional status and well-being) [27] at 4 weeks after the last dose of study drug
- Mean change from baseline (Day 0) in scores on the Migraine Disability Assessment (MIDAS; scores range from 0 to 270, with higher scores indicating more severe disability) [28, 29]
- Mean change from baseline (Day 0) in domain scores on the Migraine-Specific Quality of Life (MSQoL) questionnaire (domains assessed: role function-restrictive [RFR; 7 items on how migraine limits daily activities], role function-preventive [RFP, 4 items on how migraine prevents these activities], emotional function [EF; 3 items on the emotional effects of migraine] [30]; scores range from 0 to 100, with higher scores indicating better health-related quality of life) at 4 weeks after the last dose of study drug
- Proportion of participants classified as responders on the Patient Global Impression of Change (PGIC; responder defined as an individual who reported a rating of 5–7 [moderately better, better, or a great deal better] on the PGIC)
- Change from baseline (Day 0) in domain scores on the Work Productivity and Activity Impairment (WPAI) assessment (percent work time missed due to health, percent impairment while working due to health, percent overall work impairment due to health, percent activity impairment due to health) [31]

Adverse events (AEs), serious AEs (SAEs), AEs leading to discontinuation, and CV AEs in participants with a CV medical history were also assessed.

### Statistical analyses

Efficacy analyses were conducted in the full analysis set, which included all randomly assigned participants who received ≥1 dose of study drug and had ≥10 days of postbaseline efficacy assessments on the primary endpoint. Demographic and baseline characteristics were summarized descriptively. The mean change from baseline in the monthly average number of migraine days, weekly average number of migraine days, monthly average number of headache days of at least moderate severity, monthly average number of days of acute medication use, and MSQoL domain scores were analyzed using a mixed-effects model for repeated measures (MMRM) with treatment, sex or gender, study, region, month, and treatment-by-month as fixed effects and baseline value and years since onset of migraine as covariates. Mean changes from baseline in the monthly average number of migraine days and the monthly average number of headache days of at least moderate severity were evaluated separately for people with CM and EM. Mean changes from baseline in WPAI domain scores, HIT-6 scores, and MIDAS scores were evaluated using an analysis of covariance model with treatment, sex or gender, study, and region as fixed effects and baseline score and years since onset of migraine as covariates. The proportion of participants with a ≥ 50% reduction in the monthly average number of migraine days was evaluated using a logistic regression model with treatment, sex or gender, and region as effects. Participants who discontinued early were considered non-responders for the overall analysis and each month following discontinuation. PGIC responder rates were evaluated using a Cochran-Mantel-Haenszel (CMH) test stratified by study.

Adverse events, SAEs, AEs leading to discontinuation, and CV AEs in participants with a CV medical history were summarized descriptively.

### Data availability

Anonymized data will be shared upon request from any qualified investigator.

## Results

### Participants

A total of 2843 participants were enrolled across all 3 studies (HALO CM, *N* = 1130; HALO EM, *N* = 875; FOCUS, *N* = 838). The overall pooled full analysis population included 2823 participants (placebo, *n* = 939; quarterly fremanezumab, n = 939; monthly fremanezumab, *n* = 945). Of these, 246 (8.7%) participants were ≥ 60 years of age and were included in these analyses (placebo, *n* = 80; quarterly fremanezumab, *n* = 74; monthly fremanezumab, *n* = 92). Demographics and baseline characteristics for participants ≥60 years were similar across treatment groups. The mean age ranged from approximately 63 to 64 years, 81% to 84% of participants were female, and the mean time since initial migraine diagnosis was approximately 37 to 38 years (Table 1). Chronic migraine (60%) was more common in this pooled subpopulation aged ≥60 years than EM (40%). As expected, time since initial migraine diagnosis was longer in the subgroup of participants aged ≥60 years than in the overall pooled population due to this subpopulation’s advanced age. For participants with a CV medical history at baseline, the most commonly reported types of CV medical history were hypertension (50%–71%) and varicose vein (0%–10%; Additional file 1).

**Table 1:** Demographics and baseline characteristics of participants ≥60 years of age and overall population

	Overall pooled population			Participants ≥60 years of age		
	Quarterly fremanezumab ***n*** = 943	Monthly fremanezumab ***n*** = 954	Placebo ***n*** = 945	Quarterly fremanezumab ***n*** = 74	Monthly fremanezumab ***n*** = 92	Placebo ***n*** = 80
Age, years, mean (SD)	42.8 (11.83)	43.0 (12.09)	42.9 (12.02)	63.5 (2.96)	63.2 (3.15)	64.0 (2.83)
Female, n (%)	811 (86)	813 (85)	808 (86)	60 (81)	77 (84)	67 (84)
White, n (%)	787 (83)	804 (84)	787 (83)	71 (96)	81 (88)	70 (88)
BMI, kg/m^2^, mean (SD)	26.3 (4.98)	26.0 (4.94)	26.4 (4.81)	25.5 (4.14)	25.7 (4.18)	26.0 (4.01)
Migraine classification, n (%)						
Chronic migraine	545 (58)	553 (58)	541 (57)	56 (76)	46 (50)	45 (56)
Episodic migraine	398 (42)	401 (42)	404 (43)	18 (24)	46 (50)	35 (44)
Time since initial migraine diagnosis, years, mean (SD)	21.1 (12.77)	21.5 (12.88)	21.2 (12.92)	37.3 (13.06)	38.0 (12.31)	37.1 (12.49)

SD, standard deviation; BMI, body mass index

### Monthly migraine days, monthly headache days of at least moderate severity, and days of acute medication use

**Fig. 1:**
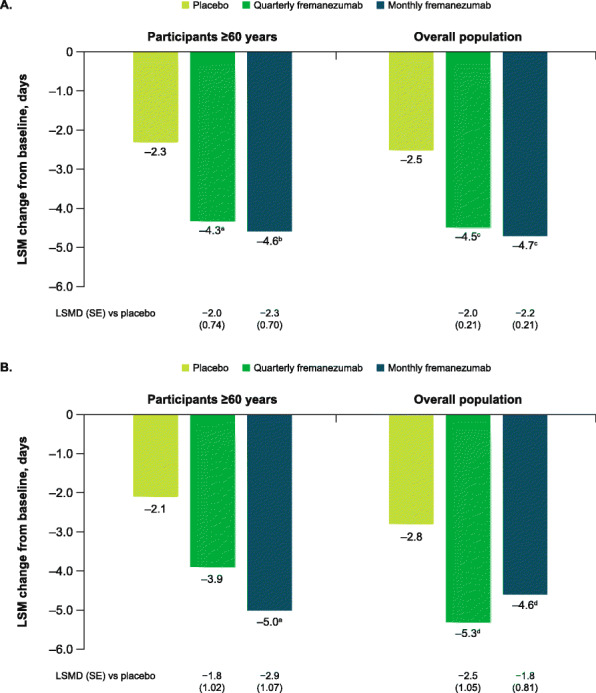
Change in monthly migraine days during 12 weeks **A**) overall and **B**) by migraine classification. Data shown are the LSM changes from baseline in the monthly average number of migraine days during 12 weeks of double-blind treatment. Part A includes data for participants ≥60 years and the overall pooled population; part B includes data for participants ≥60 years by migraine diagnosis (chronic or episodic migraine). LSM, least-squares mean; LSMD, least-squares mean difference; SE, standard error. ^a^*P* < 0.01 vs placebo. ^b^*P* < 0.005 vs placebo. ^c^*P* < 0.0001 vs placebo. ^d^*P* < 0.05 vs placebo

Among participants aged ≥60 years in the pooled analysis, fremanezumab treatment resulted in significantly greater reductions from baseline during 12 weeks of double-blind treatment in the monthly average number of migraine days compared with placebo (least-squares mean [LSM (standard error [SE])] change from baseline: placebo, − 2.3 [0.57]; quarterly fremanezumab, − 4.3 [0.59]; monthly fremanezumab, − 4.6 [0.54], both *P* < 0.01 vs placebo; Fig. 1A) Compared with placebo, reductions from baseline during 12 weeks of double-blind treatment in monthly average migraine days were also shown to be significantly greater with both quarterly and monthly fremanezumab in participants aged ≥60 years with EM (*P* < 0.05 for both quarterly and monthly fremanezumab vs placebo) and with monthly fremanezumab in participants aged ≥60 years with CM (*P* < 0.01 for monthly fremanezumab vs placebo. Fig. 1B).

**Fig. 2:**
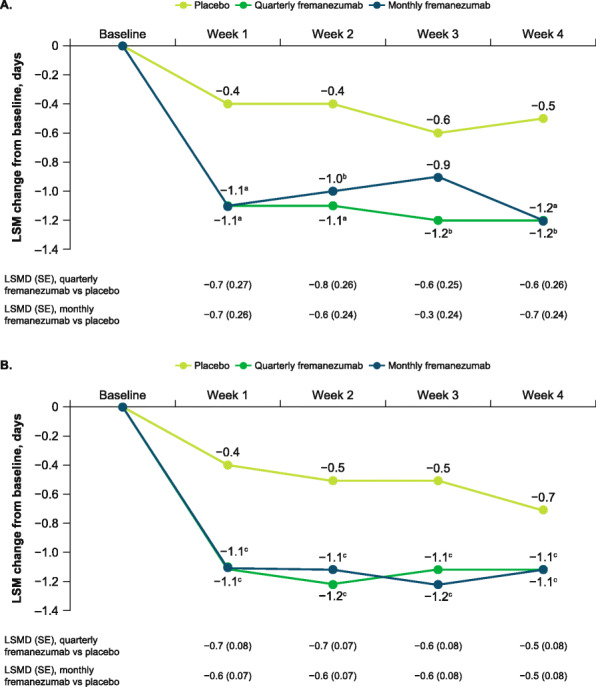
Change in weekly migraine days in **A**) participants aged ≥60 years and **B**) overall population. LSM, least-squares mean; LSMD, least-squares mean differences; SE, standard error. Data shown are the LSM changes from baseline in the weekly average number of migraine days over the first 4 weeks of double-blind treatment. ^a^*P* < 0.01 vs placebo. ^b^*P* < 0.05 vs placebo. ^c^*P* < 0.0001 vs placebo

During the first 4 weeks of the double-blind treatment period in participants aged ≥60 years, the reduction from baseline in the weekly average number of migraine days was significantly greater with fremanezumab compared with placebo at each weekly time point from Week 1 (LSM [SE] change from baseline: placebo, − 0.4 [0.20]; quarterly fremanezumab, − 1.1 [0.21]; monthly fremanezumab, − 1.1 [0.19], both *P* < 0.01 vs placebo) through Week 4 (placebo, − 0.5 [0.19]; quarterly fremanezumab, − 1.2 [0.20], *P* < 0.05 vs placebo; monthly fremanezumab, − 1.2 [0.18], *P* < 0.01 vs placebo; Fig. 2A).

**Fig. 3:**
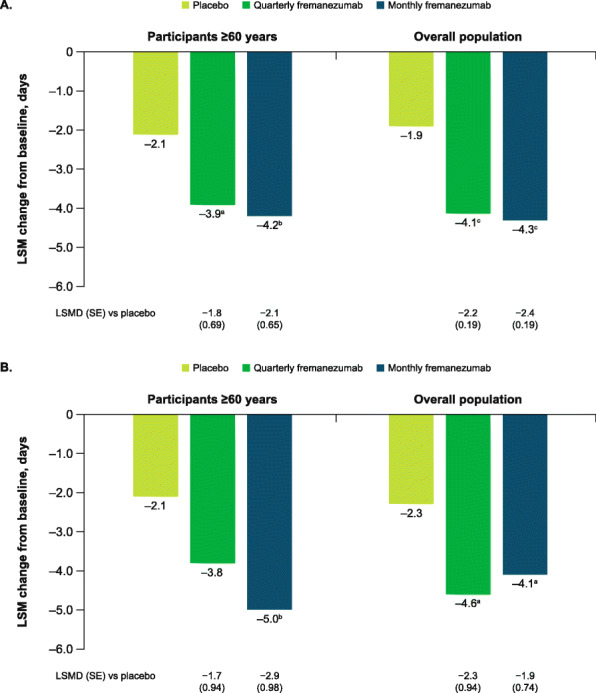
Change in monthly headache days during 12 weeks **A**) overall and **B**) by migraine classification. LSM, least-squares mean; LSMD, least-squares mean difference; SE, standard error. Data shown are the LSM changes from baseline in the monthly average number of headache days of at least moderate severity during 12 weeks of double-blind treatment. Part A includes data for participants ≥60 years and the overall pooled population; part B includes data for participants ≥60 years by migraine diagnosis (chronic or episodic migraine). ^a^*P* < 0.05 vs placebo. ^b^*P* < 0.005 vs placebo. ^c^*P* < 0.0001 vs placebo

In this pooled analysis, treatment with fremanezumab resulted in a significantly greater reduction in the monthly average number of headache days of at least moderate severity compared with placebo during 12 weeks of treatment in participants aged ≥60 years (LSM [SE] change from baseline: placebo, − 2.1 [0.53]; quarterly fremanezumab, − 3.9 [0.55], *P* < 0.05 vs placebo; monthly fremanezumab, − 4.2 [0.51], *P* < 0.01 vs placebo; Fig. 3A). Reductions in monthly average headache days of at least moderate severity from baseline during 12 weeks of double-blind treatment were significantly greater with quarterly and monthly fremanezumab compared with placebo in participants aged ≥60 years with EM (*P* < 0.05 for both quarterly and monthly fremanezumab vs placebo) and with monthly fremanezumab compared with placebo in the CM subset (*P* < 0.01 for monthly fremanezumab vs placebo; Fig. 3B).

**Fig. 4:**
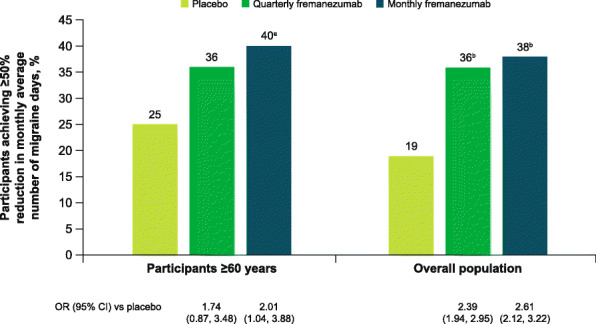
Participants achieving ≥50% response in participants aged ≥60 years and overall population. OR, odds ratio; CI, confidence interval. A ≥ 50% response was defined as a ≥ 50% reduction from baseline in the monthly average number of migraine days during 12 weeks of double-blind treatment. ^a^*P* < 0.05 vs placebo. ^b^*P* < 0.0001 vs placebo

Among participants ≥60 years of age, the proportion of participants with ≥50% reduction in the monthly average number of migraine days during 12 weeks of treatment was significantly greater with monthly fremanezumab compared with placebo (placebo, 25%; quarterly fremanezumab, 36%, *P* = 0.12 vs placebo; monthly fremanezumab, 40%, *P* < 0.05 vs placebo; Fig. 4**)**.

**Fig. 5:**
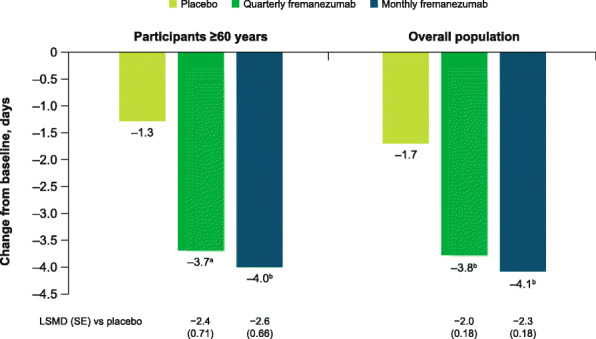
Change in monthly days of acute medication use in participants aged ≥60 years and overall population. LSM, least-squares mean; LSMD, least-squares mean difference; SE, standard error. Data shown are the LSM changes from baseline in the monthly average number of days of acute headache medication use during 12 weeks of double-blind treatment. ^a^*P* < 0.001 vs placebo. ^b^*P* ≤ 0.0001 vs placebo

During 12 weeks of treatment, quarterly and monthly fremanezumab treatment resulted in significantly greater reductions from baseline in the monthly average number of days of acute medication use compared with placebo in participants aged ≥60 years (LSM [SE] change from baseline: placebo, − 1.3 [0.55]; quarterly fremanezumab, − 3.7 [0.56], *P* < 0.001 vs placebo; monthly fremanezumab, − 4.0 [0.52], *P* = 0.0001 vs placebo; Fig. 5).

These reductions in the monthly average number of migraine days, weekly average number of migraine days, monthly average number of headache days of at least moderate severity, and monthly average number of days of acute medication use for participants aged ≥60 years were comparable to those for the overall pooled population (Figs. 1, 2B, 3, 4, and 5).

### Disability and quality-of-life outcomes

**Fig. 6:**
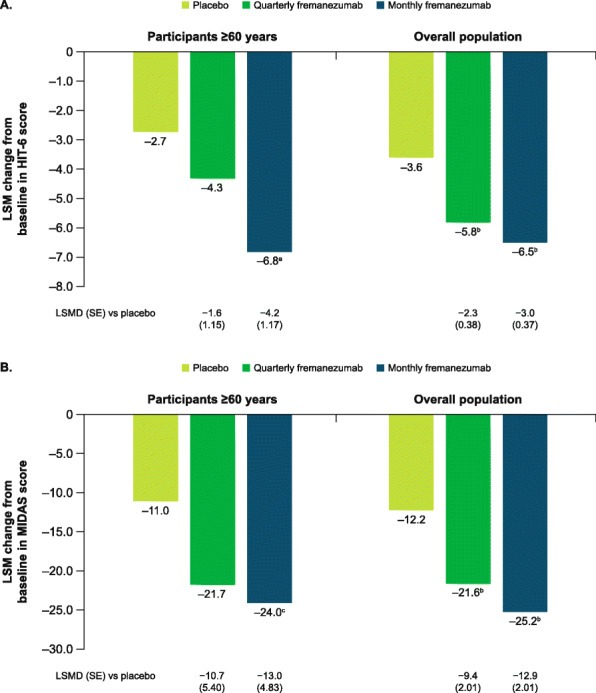
Change in **A**) HIT-6 and **B**) MIDAS scores in participants aged ≥60 years and overall population. HIT-6, 6-item Headache Impact Test; MIDAS, Migraine Disability Assessment; LSM, least-squares mean; LSMD, least-squares mean difference; SE, standard error. Data shown are the LSM changes from baseline in HIT-6 and MIDAS scores during 12 weeks of double-blind treatment. ^a^*P* = 0.0005 vs placebo. ^b^*P* < 0.0001 vs placebo. ^c^*P* < 0.01 vs placebo

During the 12-week treatment period, reductions from baseline in HIT-6 scores in participants aged ≥60 years were greater with fremanezumab versus placebo, with significant differences for monthly fremanezumab compared with placebo (LSM [SE] change from baseline: placebo, − 2.7 [0.92]; quarterly fremanezumab, − 4.3 [0.89], *P* = 0.1585 vs placebo; monthly fremanezumab, − 6.8 [0.94], *P* = 0.0005 vs placebo; Fig. 6A). The LSM difference (SE) in the change in HIT-6 score for monthly fremanezumab versus placebo (− 4.2 [1.17]) met the 2.3-point criterion for a clinically meaningful improvement [32].

Reductions from baseline in MIDAS scores during 12 weeks of treatment in participants aged ≥60 years were also greater with both fremanezumab dosing regimens compared with placebo, with a significant difference for monthly fremanezumab compared with placebo (placebo, − 11.0 [3.85]; quarterly fremanezumab, − 21.7 [4.37], *P* = 0.0506 vs placebo; monthly fremanezumab, − 24.0 [3.56], *P* < 0.01 vs placebo; Fig. 6B).

**Fig. 7:**
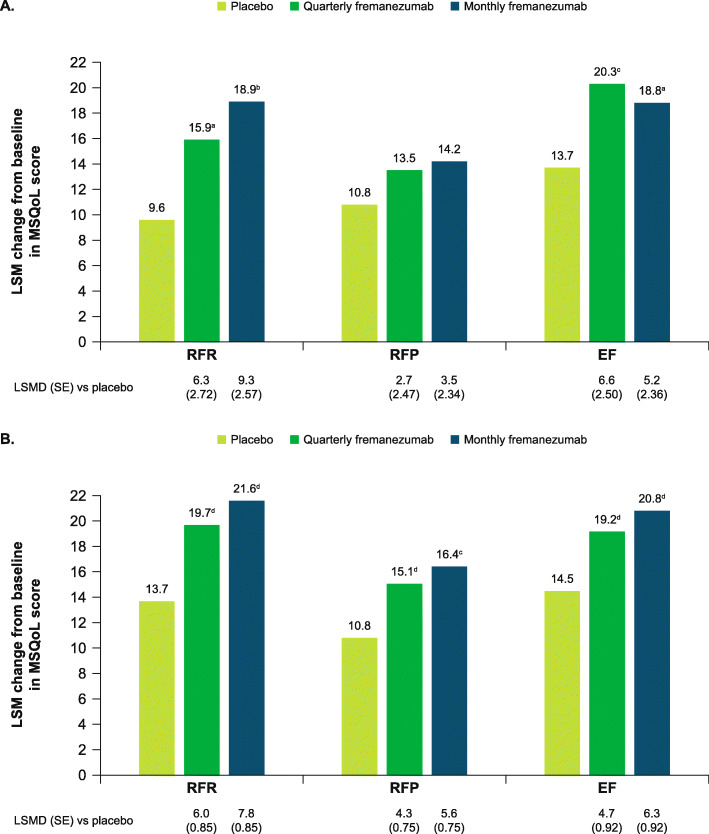
Change in MSQoL domain scores in **A**) participants aged ≥60 years and **B**) overall population. MSQoL, Migraine-specific Quality of Life; RFR, role-function restrictive; RFP, role-function preventive; EF, emotional function; LSM, least-squares mean; LSMD, least-squares mean difference; SE, standard error. Data shown are the LSM changes from baseline in MSQoL domain scores at Week 12. ^a^*P* < 0.05 vs placebo. ^b^*P* < 0.0005 vs placebo. ^c^*P* < 0.01 vs placebo. ^d^*P* < 0.0001 vs placebo

MSQoL scores improved from baseline in participants aged ≥60 years with both fremanezumab dosing regimens across all domains, including the RFR score and EF scores (Fig. 7A). These improvements in MSQoL scores at 3 months were significantly greater with both fremanezumab dosing regimens compared with placebo for participants aged ≥60 years for the RFR and EF scores (all *P* < 0.05 vs placebo).

**Fig. 8:**
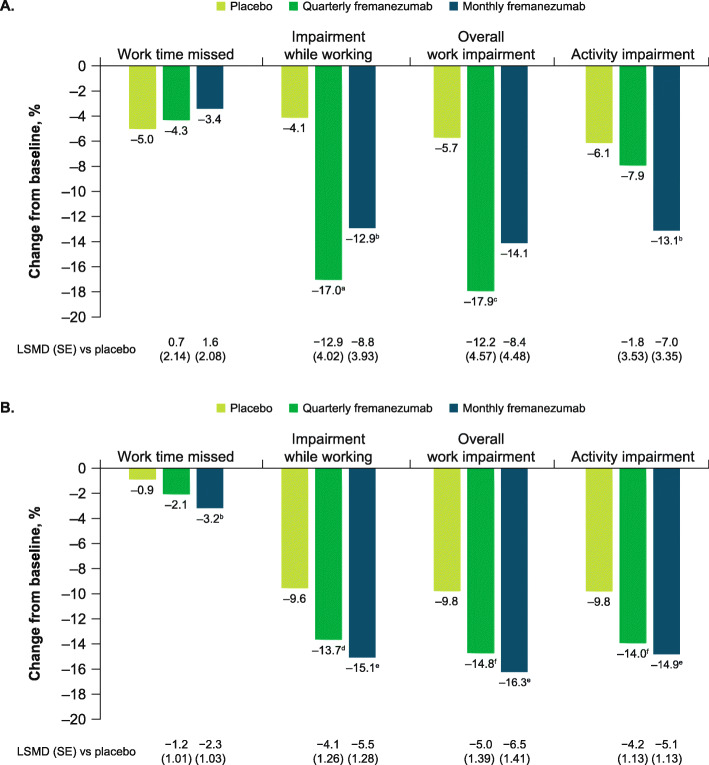
Change in WPAI domain scores in **A**) participants aged ≥60 years and **B**) overall population. WPAI, Work Productivity and Activity Impairment; LSM, least-squares mean; LSMD, least-squares mean difference; SE, standard error. Data shown are the LSM changes from baseline in WPAI domain scores during 12 weeks of double-blind treatment. ^a^*P* < 0.005 vs placebo. ^b^*P* < 0.05 vs placebo. ^c^*P* < 0.01 vs placebo. ^d^*P* ≤ 0.001 vs placebo. ^e^*P* < 0.0001 vs placebo. ^f^*P* < 0.0005 vs placebo

Improvements in all WPAI domain scores were observed in participants aged ≥60 years with fremanezumab treatment (Fig. 8A). Compared with placebo, improvements in WPAI scores were significantly greater for the percent impairment while working domain score for both fremanezumab dosing regimens, for the percent overall work impairment domain score for quarterly fremanezumab, and for the percent activity impairment score for monthly fremanezumab (all *P* < 0.05 vs placebo).

**Fig. 9:**
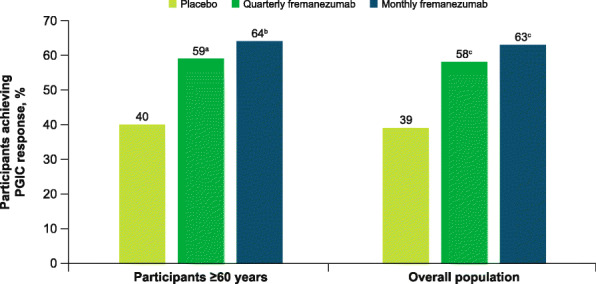
Proportion of PGIC responders in participants aged ≥60 years and the overall population. PGIC, patient global impression of change. Data shown are the proportion of PGIC responders (defined as individuals who reported a rating of 5–7 [moderately better, better, or a great deal better] on the PGIC) at Week 12. ^a^*P* < 0.01 vs placebo. ^b^*P* < 0.005 vs placebo. ^c^*P* < 0.0001 vs placebo

The proportion of participants aged ≥60 years who were classified as responders on the PGIC scale (score ≥ 5) was significantly higher with both fremanezumab dosing regimens compared with placebo (both *P* < 0.01; Fig. 9).

Improvements in HIT-6, MIDAS, MSQoL, WPAI, and PGIC scores for the subgroup of participants aged ≥60 years were comparable to those in the overall pooled population (Figs. 6, 7B, 8B, and 9).

### Safety

This pooled analysis of AEs reported in a subgroup of participants aged ≥60 years showed that fremanezumab was generally safe and well tolerated. In the overall subgroup of participants ≥60 years of age, AEs were reported for similar proportions of participants across treatment groups. Serious AEs (all treatment groups, 3%) and AEs leading to discontinuation (quarterly fremanezumab, 1%; monthly fremanezumab, 1%; placebo, 3%) were infrequent in participants receiving fremanezumab and comparable to placebo. The most common AEs were injection-site induration, injection-site pain, and injection-site erythema (Table 2). Among participants aged ≥60 years with a CV medical history, CV AEs occurred in a similar proportion of participants with or without a CV medical history (Table 3). There was only one SAE reported in participants aged ≥60 years with a CV medical history, and none reported in participants aged ≥60 years without a CV medical history. These safety results are comparable to those in the overall pooled population (any AE, quarterly fremanezumab [65%], monthly fremanezumab [62%], placebo [58%]; SAEs, quarterly fremanezumab [<1%], monthly fremanezumab [1%], placebo [2%]; AEs leading to discontinuation, quarterly fremanezumab [1%], monthly fremanezumab [2%], placebo [2%]; any CV AE with CV medical history, quarterly fremanezumab [4%], monthly fremanezumab [6%], placebo [4%]).

**Table 2:** Overall AEs in participants ≥60 years of age with ≥5% incidence in any treatment group

AEs, n (%)	Quarterly fremanezumab (*n* = 74)	Monthly fremanezumab (*n* = 92)	Placebo (*n* = 80)
Any AE	48 (65)	53 (58)	44 (55)
SAEs	2 (3)	3 (3)	2 (3)
AEs leading to discontinuation	1 (1)	1 (1)	2 (3)
Most common AEs (incidence ≥5% in any treatment group)			
Injection-site induration	11 (15)	23 (25)	14 (18)
Injection-site pain	13 (18)	19 (21)	10 (13)
Injection-site erythema	8 (11)	10 (11)	13 (16)
Nasopharyngitis	4 (5)	5 (5)	4 (5)

AE, adverse event; SAE, serious adverse event

**Table 3:** Cardiovascular adverse events in participants ≥60 years of age with a cardiovascular medical history

CV AEs, n (%)	Quarterly fremanezumab	Monthly fremanezumab		Placebo
	(CM/EM: 675 mg/PBO/PBO)	(EM: 225/225/225 mg)	(CM: 675/225/225 mg)	
***Participants with CV medical history***	***(n = 30)***	***(n = 6)***	***(n = 14)***	***(n = 23)***
≥1 CV AE	1 (3)	0	2 (14)	1 (4)
**AEs with incidence ≥1 participant in any treatment/dose group**				
Palpitations	0	0	1 (7)	1 (4)
Hypertensive crisis	0	0	1 (7)	0
Supraventricular tachycardia	1 (3)	0	0	0
***Participants without CV medical history***	***(n = 44)***	***(n = 40)***	***(n = 32)***	***(n = 57)***
≥1 CV AE	0	0	2 (6)	0
**AEs with incidence ≥1 participant in any treatment/dose group**				
Hot flush	0	0	1 (3)	0
Hypertension	0	0	1 (3)	0

CM, chronic migraine; EM, episodic migraine; PBO, placebo; CV, cardiovascular; AE, adverse event

## Discussion

As compared with placebo, fremanezumab treatment resulted in reductions of approximately 2 days from baseline in the monthly average number of migraine days and in the monthly average number of headache days of at least moderate severity over 12 weeks in this subgroup of participants ≥60 years of age. Reductions in migraine days were seen as early as Week 1 with fremanezumab treatment, and consistent significant reductions were observed with both fremanezumab dosing regimens compared with placebo during each of the first 4 weeks of treatment. The proportion of participants achieving clinically meaningful response rates (≥50% reduction in monthly average number of migraine days) with fremanezumab was 11% higher with quarterly dosing and 15% higher with monthly dosing compared with placebo; the difference between fremanezumab and placebo was statistically significant for monthly fremanezumab, but not quarterly fremanezumab. Additionally, over 12 weeks of fremanezumab treatment, participants aged ≥60 years experienced an approximately 2.5-day per month greater reduction in days of acute headache medication use than with placebo. These results were comparable to those observed in the overall pooled population.

In participants aged ≥60 years, treatment with quarterly or monthly fremanezumab over 12 weeks resulted in clinically meaningful improvements in a variety of outcomes related to quality of life. Participants receiving fremanezumab experienced significantly greater improvements in headache-related disability, based on reductions in MIDAS and HIT-6 scores, as compared with participants who received placebo. Improvements from baseline versus placebo were observed with fremanezumab in all MSQoL and WPAI domains. The proportion of participants categorized as PGIC responders was also significantly greater with fremanezumab versus placebo.

Although migraine treatment may be more difficult in older patients [5, 7], fremanezumab showed comparable, or for some endpoints numerically better, efficacy in the subgroup of participants aged ≥60 years and in the overall pooled population. For example, for the WPAI domain of work time missed, fremanezumab showed numerically greater improvements from baseline in the subgroup aged ≥60 years compared with the overall population. This consistency of the efficacy of fremanezumab in this subgroup of participants aged ≥60 years with that in the overall pooled population provides evidence that fremanezumab is effective in reducing migraine in older individuals, including those with difficult-to-treat migraine.

Determining an optimal migraine preventive pharmacotherapy plan for older patients can be challenging due to the traditionally lower participation rates by older people with migraine, as well as restrictive exclusion criteria in most migraine clinical trials [5]. The safety profile for fremanezumab in participants aged ≥60 years is consistent across treatment groups and is comparable to the overall pooled population. Generally, AEs were infrequent in older participants with a CV medical history. Of note, considering the higher risk for CV disease and other comorbidities among older individuals [9, 33], rates of CV AEs were low, even in participants with a CV medical history. Further, there were no SAEs reported in this subgroup.

Whereas migraine is more common in individuals younger than 55 years, a considerable proportion of people older than this also experience migraine [7, 9, 33]. Many cross-sectional studies have shown that migraine persists in people aged ≥60 years, with a prevalence of approximately 5% even in those older than 75 years [34]. Currently, there are no subgroup analyses for other mAbs targeting the CGRP ligand that examined efficacy in older study participants with migraine. Erenumab is the only other CGRP pathway-targeted mAb (receptor targeted) that has analyzed safety and tolerability in subgroups of older study participants (50–55 and >55 years of age) with migraine. As with fremanezumab, treatment-emergent AE rates were comparable for the erenumab treatment groups and the placebo group in these older subgroups [35]. Rates of SAEs and AEs leading to discontinuation were also infrequent and similar across the erenumab and placebo groups for these older participants [35].

The results of this subgroup analysis may be subject to limitations. Although this analysis included participants from 3 large studies, there were relatively small numbers of participants ≥60 years in each group. The lower number of participants may have limited the ability to detect significant between-group differences for all endpoints (eg, the ≥50% response rate for change in monthly migraine days for quarterly fremanezumab versus placebo). However, the results reported here may be generalizable because the efficacy and safety results from the subgroup were comparable to the overall pooled population. Additionally, of 7171 participants screened in the HALO and FOCUS studies, 610 met the exclusion criteria, which included a history of clinically significant CV disease as determined by the investigator, so the participants included in these analyses likely have a milder CV medical history than older people with migraine in the general population.

As mentioned, older individuals with migraine likely have a range of comorbidities, including hypertension, heart disease, or psychiatric disorders, that also influence management of their migraine [7, 33]. Even in a complex population, effective migraine management may improve their quality of life [7, 33]. With a favorable safety profile, these results demonstrate that fremanezumab may be an effective migraine preventive treatment option for older individuals with difficult-to-treat disease.

## Conclusions

This pooled subgroup analysis demonstrated that fremanezumab treatment was effective in reducing migraine days, headache days of at least moderate severity, and days of acute medication use over 12 weeks of treatment in participants aged ≥60 years with EM or CM. Additionally, fremanezumab improved quality of life and headache-related disability in this older population. The consistent efficacy and safety results of fremanezumab across treatment groups in this subgroup analysis demonstrate that fremanezumab is an effective preventive treatment option for individuals ≥60 years of age, including those with comorbidities and difficult-to-treat EM or CM.

## Supplementary Information

**Additional file 1.** Types of CV Medical History at Baseline.

## Data Availability

Anonymized data, as described in this manuscript, will be shared upon request from any qualified investigator by the author investigators or Teva Pharmaceuticals Industries, Ltd.

## Abbreviations

CGRP: calcitonin gene–related peptide
CM: chronic migraine
CV: cardiovascular
ECG: electrocardiogram
EM: episodic migraine
mAbs: monoclonal antibodies
ICHD: International Classification of Headache Disorders
HIT-6: 6-item Headache Impact Test
MIDAS: Migraine Disability Assessment
RFP: role function-preventive
RFR: role function-restrictive
EF: emotional function
WPAI: Work Productivity and Activity Impairment
PGIC: Patient Global Impression of Change
AE: adverse event
SAE: serious adverse event
LSM: least-squares mean
LSMD: least-squares mean difference
OR: odds ratio
BMI: body mass index
PBO: placebo
