# Correction: Efficacy and safety of fremanezumab in clinical trial participants aged ≥60 years with episodic or chronic migraine: pooled results from 3 randomized, double-blind, placebo-controlled phase 3 studies

**DOI:** 10.1186/s10194-022-01423-x

**Published:** 2022-05-17

**Authors:** Stephanie J. Nahas, Steffen Naegel, Joshua M. Cohen, Xiaoping Ning, Lindsay Janka, Verena Ramirez Campos, Lynda J. Krasenbaum, Dagny Holle-Lee, David Kudrow, Christian Lampl

**Affiliations:** 1 Department of Neurology, Thomas Jefferson University, Philadelphia, PA USA; 2 Department of Neurology, University Hospital Halle (Saale) and University Halle-Wittenberg, Halle, Germany; 3 Teva Pharmaceutical Industries, West Chester, PA USA; 4 Department of Neurology and Westgerman Headache Center Essen, University Hospital, Essen, Germany; 5 California Medical Clinic for Headache, Santa Monica, CA USA; 6 Headache Medical Centre, Linz, Austria; 7 Department of Neurology, Konventhospital Barmherzige Brüder, Linz, Austria

**Correction to: J Headache Pain 22, 141 (2021)**

**https://doi.org/10.1186/s10194-021-01351-2**

Following the publication of the original article [1], we were notified that the x-axis labels for part B of Figure 1 were incorrect in the previous version and had accidentally been duplicated from part A.

Originally published Figure 1:
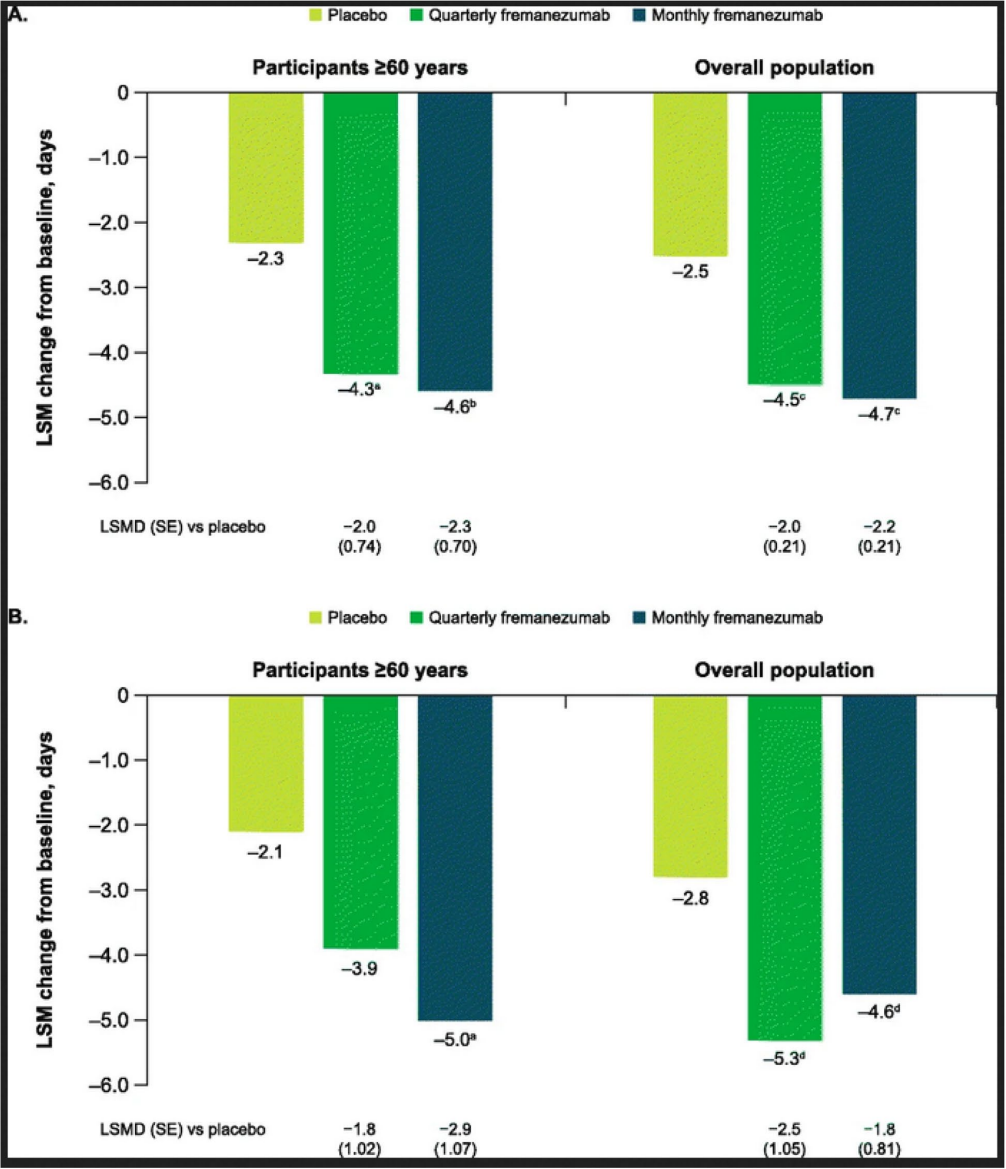


Corrected Figures:
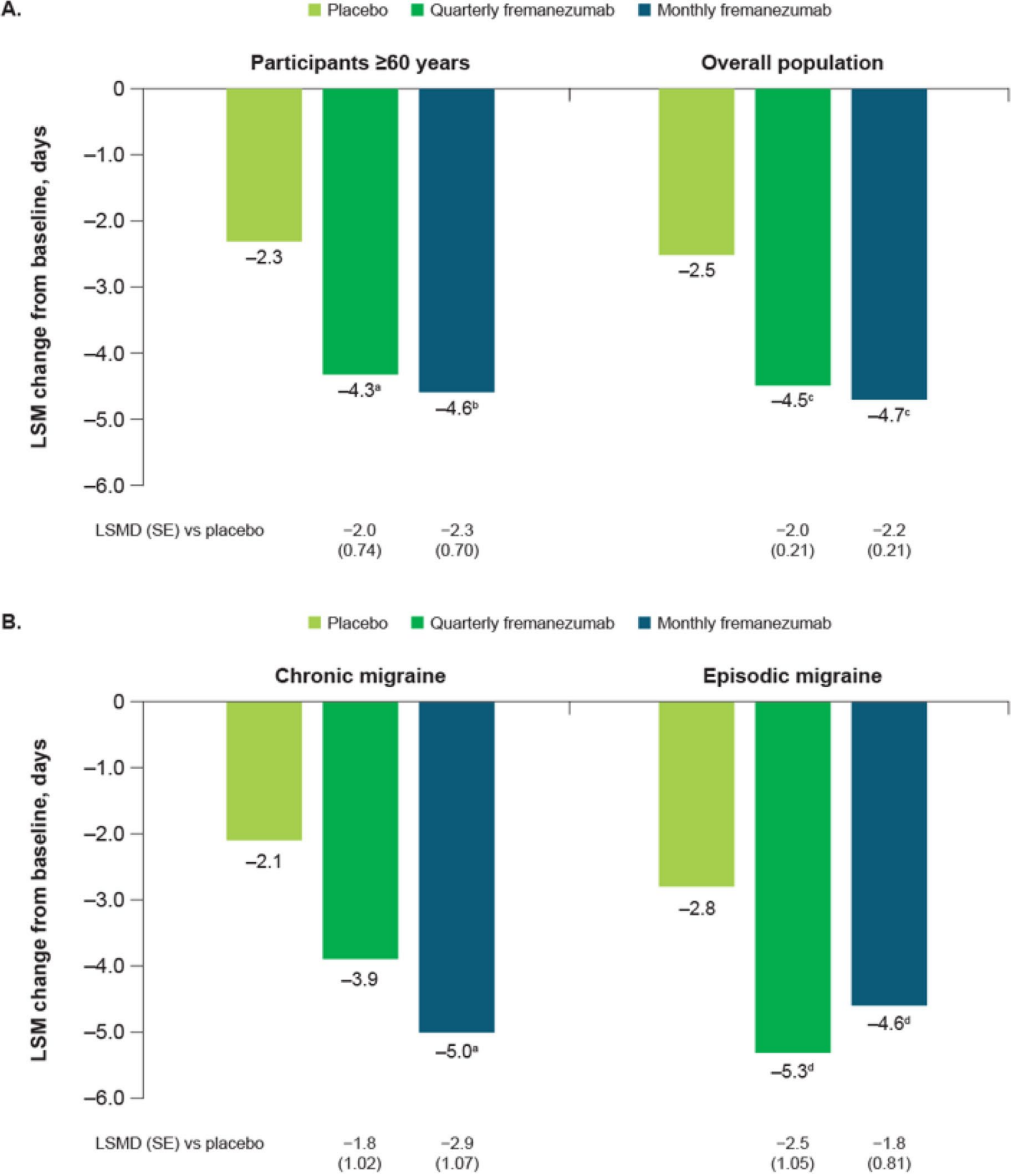


The original article has been corrected.
