# Supplementary material for: Efficacy and safety of fremanezumab in clinical trial participants aged ≥60 years with episodic or chronic migraine: pooled results from 3 randomized, double-blind, placebo-controlled phase 3 studies
Source: J Headache Pain. 2021 Nov 24;22(1):141. doi: 10.1186/s10194-021-01351-2 (PMC8903616; doi:10.1186/s10194-021-01351-2)
Supplement: Supplementary file 1 — Additional file 1. Types of CV Medical History at Baseline. [file 10194_2021_1351_MOESM1_ESM.docx]

**Additional File 1. Types of CV Medical History at Baseline^a^**

| **CV medical history, n (%)^b^** | **Quarterly fremanezumab**  **(CM/EM: 675 mg/PBO/PBO)**  **(n = 30)** | **Monthly fremanezumab**  **(EM: 225/225/225 mg)**  **(n = 6)** | **Monthly fremanezumab**  **(CM: 675/225/225 mg)**  **(n = 14)** | **PBO**  **(n = 23)** |
| --- | --- | --- | --- | --- |
| Hypertension | 18 (60) | 3 (50) | 10 (71) | 14 (61) |
| Varicose vein | 3 (10) | 0 (0) | 1 (7) | 2 (9) |
| Hot flush | 0 (0) | 2 (33) | 1 (7) | 0 (0) |
| Raynaud phenomenon | 3 (10) | 0 (0) | 0 (0) | 1 (4) |
| Atrial fibrillation | 0 (0) | 2 (33) | 0 (0) | 0 (0) |
| Mitral valve incompetence | 2 (7) | 0 (0) | 0 (0) | 0 (0) |
| Mitral valve prolapse | 2 (7) | 0 (0) | 0 (0) | 0 (0) |
| Supraventricular tachycardia | 2 (7) | 0 (0) | 0 (0) | 1 (4) |
| Irregular heart rate | 2 (7) | 0 (0) | 0 (0) | 0 (0) |
| Cardiac ablation | 2 (7) | 0 (0) | 0 (0) | 0 (0) |

CV, cardiovascular; CM, chronic migraine; EM, episodic migraine; PBO, placebo.

^a^Percentages are reported based on the number of participants with CV medical history at baseline.

^b^Types of CV medical history reported for >1 participant in any treatment group. Additional types of CV medical history reported for ≤1 participant in any treatment group included first-degree atrioventricular block, left bundle-branch block, myocardial infarction, palpitations, sinus bradycardia, bradycardia, pericardial effusion, vertebral artery hypoplasia, cardiac murmur, angiogram, decreased ejection fraction, intracranial aneurysm, implantable cardiac monitor insertion, deep vein thrombosis, hematoma, hypotension, labile blood pressure, and lymphedema.
